# Intensive field measurements for characterizing the permeability and methane release with the treatment process of pressure-relief mining

**DOI:** 10.1038/s41598-022-19283-5

**Published:** 2022-09-01

**Authors:** Cun Zhang, Ziyu Song, Qingsheng Bai, Lei Zhang, Jianhang Chen

**Affiliations:** 1 Beijing Key Laboratory for Precise Mining of Intergrown Energy and Resources, China University of Mining and Technology (Beijing), Beijing, 100083 China; 2 Key Laboratory of Deep Coal Resource Ming, Ministry of Education, China University of Mining and Technology, Xuzhou, 221116 China; 3 Geotechnical Institute, TU Bergakademie Freiberg, Gustav-Zeuner-Straße 1, 09599 Freiberg, Germany; 4 School of Mines, China University of Mining & Technology, Xuzhou, 221116 Jiangsu China

**Keywords:** Environmental sciences, Natural hazards, Energy science and technology, Engineering

## Abstract

Characterizing the permeability evolution and methane release is of great significance for the safe mining of the high gas outburst seams, as well as coal and gas simultaneous extraction. It contributes to reduce methane emissions from coal mining for greenhouse effect control. Theoretical analysis, laboratory testing, and numerical simulation are widely used methods to characterize the permeability and methane release with the treatment process of pressure-relief mining. However, these methods cannot fully reflect the complexity of filed practice. In this study, we report the effectiveness of protective coal seam (PCS) mining and the pressure-relief area in the protected coal seam (PDCS) based on detailed and integrated field measurements in a Chinese coal mine. To the best of our knowledge, it is the first time to measure the permeability coefficient and gas pressure evolution in the PDCS during the process of PCS longwall mining. The evolution of the permeability coefficient in the pressure-relief area during PCS mining can be divided into four stages: slowly decreasing, sharply increasing, gradually decreasing, and basically stable. The maximum permeability coefficient is 322 times of the initial value and stabilized at 100 times after the goaf compacted. The gas pressure evolution in the PDCS indicates that the strike pressure relief angle is 52.2° at the active longwall face zone, and 59.3° at the installation roadway side. The inclined pressure relief angles at the lower and upper sides of the longwall face are 75° and 78.9°, respectively. The residual gas content and gas pressure of the PDCS in the pressure-relief area are reduced to less than 6 m^3^/t and within 0.4 MPa, respectively. The field measurements further prove that pressure-relief mining can prevent coal and gas outbursts in PDCSs. The field observations in this paper can serve as benchmark evidence for theoretical analysis and numerical simulations, and also provide insights into realizing safety mining in similar conditions.

## Introduction

Coal mine gas disaster is one of the main disasters in underground coal mining, the annual death toll from gas disasters in China accounts for more than 30% of the total deaths in coal mines^1^. However, coal seam methane is also a resource and greenhouse gas. If it is simply discharged, it will not only waste resources, but also aggravate the greenhouse effect^2,3^. Pressure-relief mining combined with three-dimensional gas drainage technology has been widely used to remove gas and coal outbursts and other dynamic disasters in China^4–6^. Many experts have investigated the protective coal seams (PCSs) pressure-relief mining and established its technical and theoretical system^7–9^. However, the pressure-relief gas from the protected coal seam (PDCS) may enter the PCS longwall face and the goaf cause a gas explosion^10–12^. Thus, understanding the permeability evolution and gas migration path in the protected coal seam (PDCS) is fundamental for the drainage of pressure-relief gas and the safe mining of the PCS. At present, numerical simulations and field observations are the main approaches to characterize the evolution of permeability and gas migration paths in surrounding rock mass.

The slug method is commonly used to assess the permeability of surrounding rocks and the characteristics of fracture development, especially in saturated coal seams^13,14^. Moreover, pumping and flow meter tests are also widely employed to evaluate the permeability of overlying rocks^15,16^. Three geophysical approaches (the flow meter test, color television observation, and the transient electromagnetic method) have been employed to detect fracture development in overlying strata^17^, in which color television is considered the best one. Using physical modeling and the slug method, Zhang and Wang^18^ demonstrated the development of induced fractures in overlying strata. Wang et al.^19^ studied the influence of mining-induced stress on the permeability of the fracture zone. However, most studies focused on the fracture development instead of the permeability evolution in the above field measurement. The evolution of permeability is mainly obtained through laboratory tests^20–23^. However, due to the heterogeneity (i.e., stress and physical parameters) of the coal mass, laboratory tests cannot fully reflect the reality of the field environment. Moreover, field measurements are usually executed after longwall mining; thus cannot predict the whole evolution of permeability in the surrounding rock mass, which is essential for the predesign of gas drainage.

**Figure 1:**
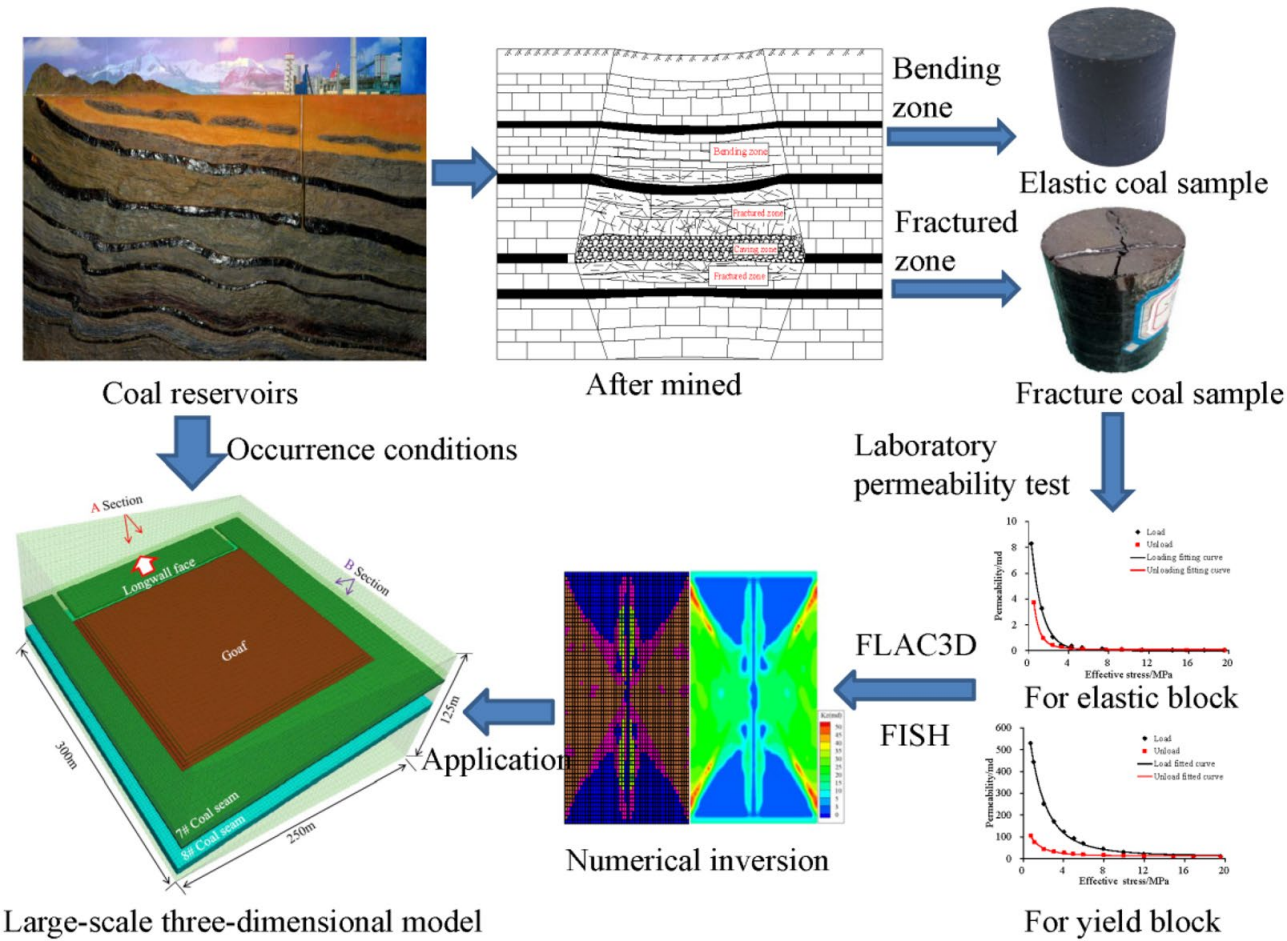
A numerical method to simulate gas migration and permeability evolution in pressure-relief mining^24^.

The relationship between the stress and permeability in the disturbed zones (i.e., bending zone, fractured zone, and caved zone) has been summarized by laboratory tests in several studies^24–28^, making it possible to simulate the permeability changes during mining and to evaluate the effectiveness of gas extraction. As shown in Fig. 1, considering disturbed zone-dependent permeability models, people could well simulate the permeability change of PDCS during longwall retreating. The permeability calculation results can be imported into seepage calculation software for seepage calculation. This method provides a promising way to evaluate the effectiveness of pressure relief and permeability enhancement in the PDCS^24,27,29,30^. However, the permeability models in the simulation are obtained from specific research areas and only describe the stress sensitivity in the loading regimes, it is debatable to utilize these models without calibrations. Thus, the numerical results are poorly verified by field applications, doubting the reliability of the simulations.

In this study, we performed intensive field measurements to characterize the permeability enhancement and the associated pressure relief regions in the PDCS during longwall retreating in the above PCS. Different from previous studies, which usually test these parameters after the PCS mining, we continually monitor the parameters mentioned above, enabling us to summarize the whole evolution of the permeability and the associated pressure-relief effectiveness during the PCS minging. Five groups of testing boreholes are arranged in PDCS to monitor the gas pressure, gas content, and permeability coefficient. The gas pressure was measured by the active pressure measurement (APM) method; the gas content was measured by the high-pressure adsorption (HPA) method; and the permeability coefficient was measured by the radial flow permeability (RFP) method. To the best our knowledge, it is the first time to investigate the permeability and gas pressure evolution of the PDCS during the PCS mining process. Moreover, regional measurements, instead of point measurements, were carried out in this work; thus, spatio-temporal evolutions of these parameters can be characterized. The outputs in this study provide fundamental insights into how permeability and gas pressure evolution of PDCS during the PCS mining process. The results can also serve as benchmarking evidence for field-scale pressure-relief mining numerical simulation and lab-scale permeability tests.

## Area of research

**Figure 2:**
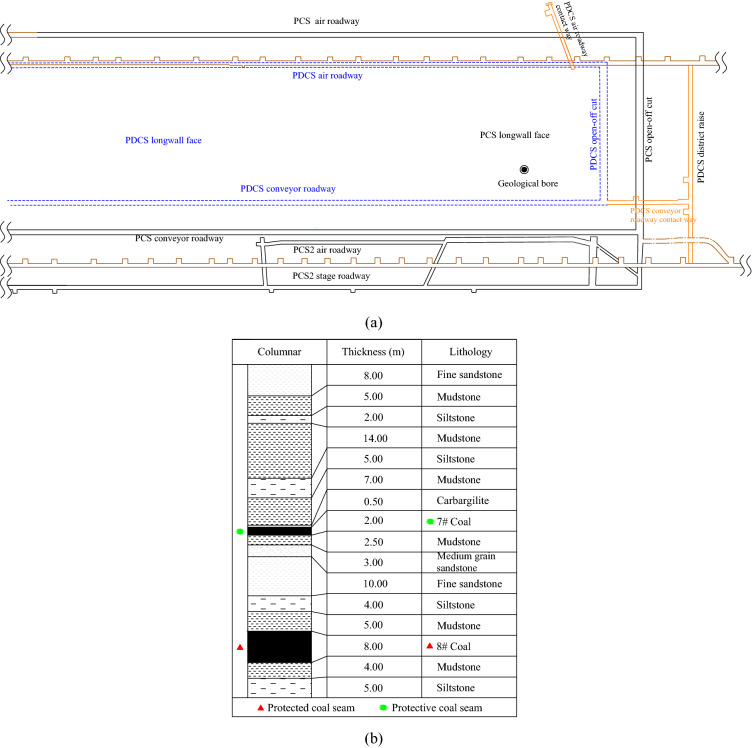
Location of PCS and PDCS longwall face at the coal mine (**a**) and borehole diagram of coal measures (**b**).

The coal seams 7^#^ and 8^#^ in the Huaibei mining area, Anhui province, are outburst tendency. The 7^#^ coal seam was mined as the upper PCS to prevent coal and gas outbursts in the 8^#^ coal seam. The average thickness of the 7^#^ coal seam is 2.0 m with a 527 m burial depth. The 8^#^ coal seam has an average thickness of 8.0 m and is located below the 7^#^ coal seam, at an average distance of 24.5 m. The relative position of the coal seam and the lithology of the roof and floor are shown in Fig. 2. Field measurements show that the average and maximum coal gas pressure of the 7^#^ coal seam are 0.8 MPa and 1.70 MPa, respectively, and the average gas content is 8.78 m^3^/t. The average gas content is 8.56 m^3^/t, and the average and the maximum gas pressure of the 8^#^ coal seam are 1.1 MPa and 1.51 MPa, respectively. The width of the first longwall face in 7^#^ coal seam (PCS 726) is 190 m, and the advancing length is 680 m. The relevant parameters of the 8^#^ coal seam are listed in Table 1.

**Table 1:** Proximate analysis and adsorption parameters of the 8^#^ coal seam.

Coal seam	M_ad_ (%)	A_ad_ (%)	V_ad_ (%)	*a* (mL/g)	*b* (MPa^−1^)
8^#^	0.94	18.32	23.56	28.3	0.88

Mad, Aad and Vad are the abbreviation of moisture air-dried basis, ash air-dried basis and volatiles air-dried basis, *a* and *b* are adsorption constants.

## Field measurement methods

### Gas pressure measurement

**Figure 3:**
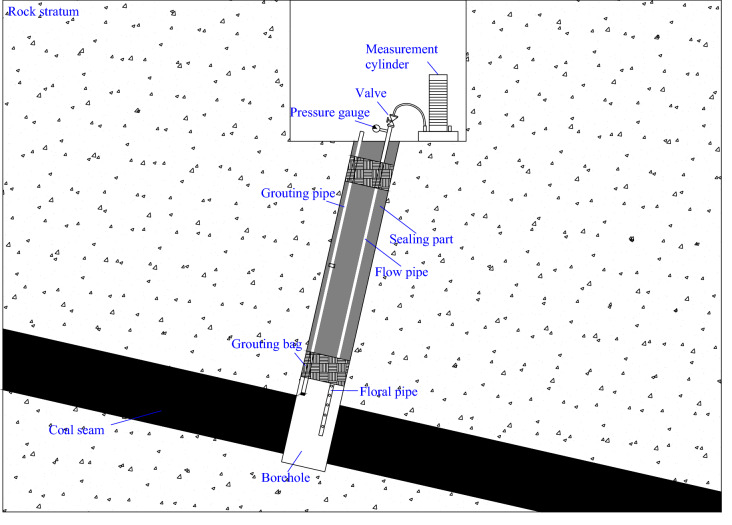
Diagram of field measurement of the gas pressure and the permeability coefficient.

This study adopts the APM method to measure the coal seam gas pressure. All the measurement holes are downward to the PDCS (Fig. 3). The cement slurry seals the hole and prevents the leakage of gas during the tests, thus ensuring the accuracy of the measurements. The arrangement of the test boreholes and sealing method are shown in Fig. 3. The process for the measurement are as follows: pipe laying → grouting and sealing → sealing the top orifice → checking the gas flow path → installing the pressure gauge → observing the gas pressure. The specific operation for each step includes: (1) Using galvanized iron pipe with pressure measuring kit (measure gas pressure), and choosing a pressure gauge with appropriate range according to the estimated pressure. (2) Pouring sodium silicate into the hole and sealing the hole with expanded cement at the same time. (3) The prepared expansive cement slurry is rapidly injected into the borehole and stopped at the location of 0.5 m to the top orifice. (4) Connecting the iron pipe with the pressure air pipe, slowly opening the pressure air, and observing whether there is airflow from the gas outlet of the pressure kit. (5) Installing pressure gauge after cement slurry has solidified for 24 h. (6) Regularly observing the gas pressure until it does not change. Due to water flows into the boreholes have an impact on the measurement results, we will ensure that the water in the borehole is drained during monitoring.

### Gas content measurement

The HPA method with direct gas content (DGC) meter is used to measure the coal seam gas content^31^. The gas content is mainly measured by underground coring, underground desorption, coal sample weighing and crushing, moisture measurement, and other methods. After drilling into the coal seam, the drill bit is withdrawn, and the coring bit is replaced. The obtained coal sample is loaded a tank immediately and sealed. Sample desorption is carried out in the site, and the data is recorded. Finally, the sealed coal sample tank is brought to the laboratory for further desorption to obtain the accurate gas content.

### Permeability coefficient measurement

**Figure 4:**
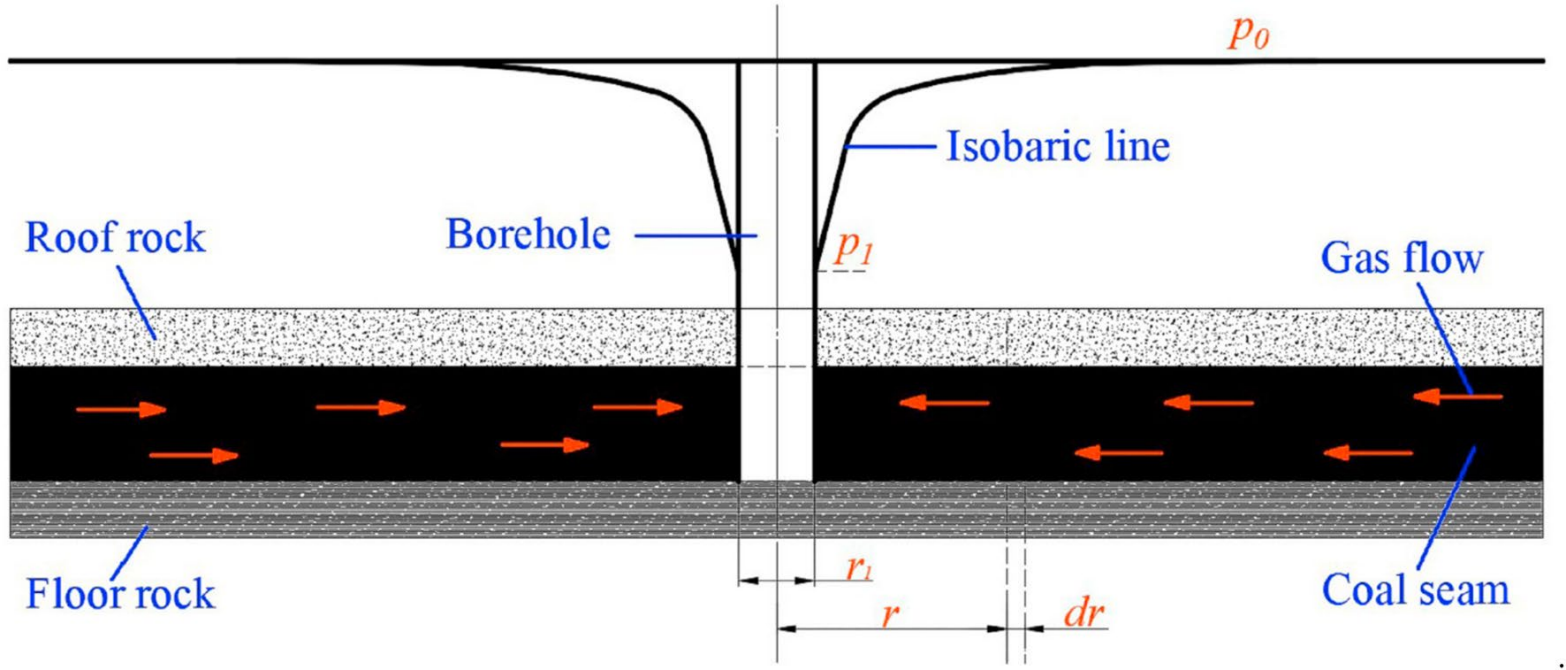
Distribution of radial flow gas pressure in homogeneous coal seam (modified after^33^).

The permeability coefficient is usually used to describe the fluidity of the gas flow in the coal seam. Over the past few decades, various methods have been proposed to measure and calculate the permeability coefficient. These methods can be divided into two categories: (a) laboratory measurement methods and (b) in-situ measurement methods. Due to the heterogeneity (i.e., stress and physical parameters) of the coal mass, laboratory tests cannot fully reflect the reality of the field environment. Radial flow permeability method is widely used in Chinese coal mines^32,33^, which is also used in this study. As shown in Fig. 4. The differential equation of the radial unsteady flow of a borehole can be given:

1 $$ \frac{\partial P}{{dt}} = \frac{{4\lambda P^{ - 0.75} }}{\alpha }\left( {\frac{{\partial^{2} P}}{{\partial r^{2} }} + \frac{1}{r} \cdot \frac{\partial P}{{\partial r}}} \right) $$

where *r* is the radius to the borehole, m; *P* is the square of the gas pressure in the coal seam, MPa^2^; *α* is the gas content coefficient, m^3^/(m^3^ MPa^1/2^); *t* is the time of gas flow, d; *λ* is the permeability coefficient of the coal seam, m^2^/(MPa^2^ d), it can also be calculated

2 $$ \lambda = \frac{k}{{2\mu p_{n} }} $$

where *p*_n_ is the atmospheric pressure in the site, MPa;* μ* is the dynamic viscosity of methane, Pa s; *k* is the permeability, m^2^. After Laplace transform and combined with the similarity theory, *λ* can be determined using the following to equations

3 $$ Y = a_{1} F_{0}^{{b_{1} }} $$

4 $$ Y = \frac{{qr_{1} }}{{\lambda (p_{0}^{2} - p_{1}^{2} )}} $$

5 $$ F_{0} = \frac{{4\lambda tp_{0}^{1.5} }}{{\alpha r_{1}^{2} }} $$

where *F*_0_ is dimensionless time criterion; *Y* is dimensionless flow criterion; *a*_1_ and *b*_1_ are regression coefficient; *α* is the gas content coefficient, m^3^/(m^3^⋅MPa^1/2^); *p*_1_ and *p*_0_ are the gas pressure in the borehole and the initial coal seam, MPa; *r*_1_ is the radius of the borehole, m; *q* is the fluid velocity at the time *t*, m^3^/(m^2^ d), i.e.,

6 $$ q = \frac{Q}{{2\pi r_{1} L}} $$

where *L* is the length of the borehole in the coal seam, generally taken as the seam thickness, m; *Q* is the borehole flow rate when the discharge time is t, m^3^/d. Let

7 $$ A = \frac{{qr_{1} }}{{p_{0}^{2} - p{}_{1}^{2} }} $$

8 $$ B = \frac{{4tp_{0}^{1.5} }}{{\alpha r_{1}^{2} }} $$

Table 2 lists the formulas for permeability coefficient calculating by the RFP method are listed in. When calculating the permeability coefficient, any formula in Table 2 can be used to obtain an assessed value first, then the results are checked by *F*_0_ = *Bλ* until the value of *F*_0_ is in the range of the selected formula.

**Table 2:** Calculation parameters of permeability coefficient by RFP method.

Dimensionless time criterion	Permeability coefficient
*F*_0_ = 10^–2^–1	*λ* = *A*^1.61^*B*^0.61^
*F*_0_ = 1–10	*λ* = *A*^1.39^*B*^0.39^
*F*_0_ = 10–10^2^	*λ* = 1.10*A*^1.25^*B*^0.25^
*F*_0_ = 10^2^–10^3^	*λ* = 1.83*A*^1.14^*B*^0.14^
*F*_0_ = 10^3^–10^5^	*λ* = 2.10*A*^1.11^*B*^0.11^
*F*_0_ = 10^5^–10^7^	*λ* = 3.14*A*^1.07^*B*^0.07^

The steps to calculate the permeability coefficient are as follows: (1) a borehole through the coal seam is drilled, then seal the borehole and measure the initial gas pressure. (2) then open the valve and reduce the pressure to the atmospheric pressure. (3) Measure the natural gas emission from the borehole two hours at a time, twice a day, and then calculate the permeability coefficient according to formulas in Table 2. Permeability coefficient tests are performed after the gas pressure measurement at the same test borehole, as shown in Fig. 3. Because of the damage near the borehole, the gas flow rate usually presents strong fluctuations during the first few hours. However, it rapidly reduces and remains constant after one day. Field operations in this study suggest that the gas flow rate is more reliable two days after the drilling.

### Test drilling holes layout

**Figure 5:**
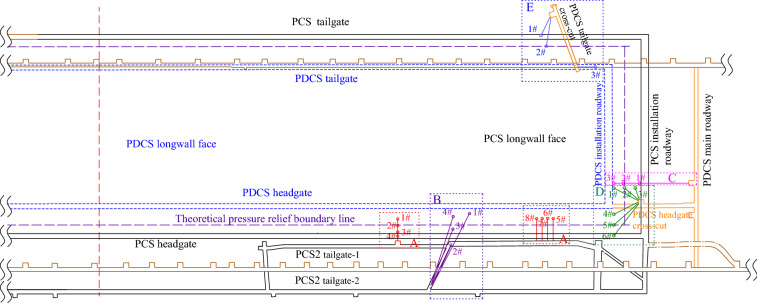
Layout of the test drilling holes in the protected coal seam.

**Figure 6:**
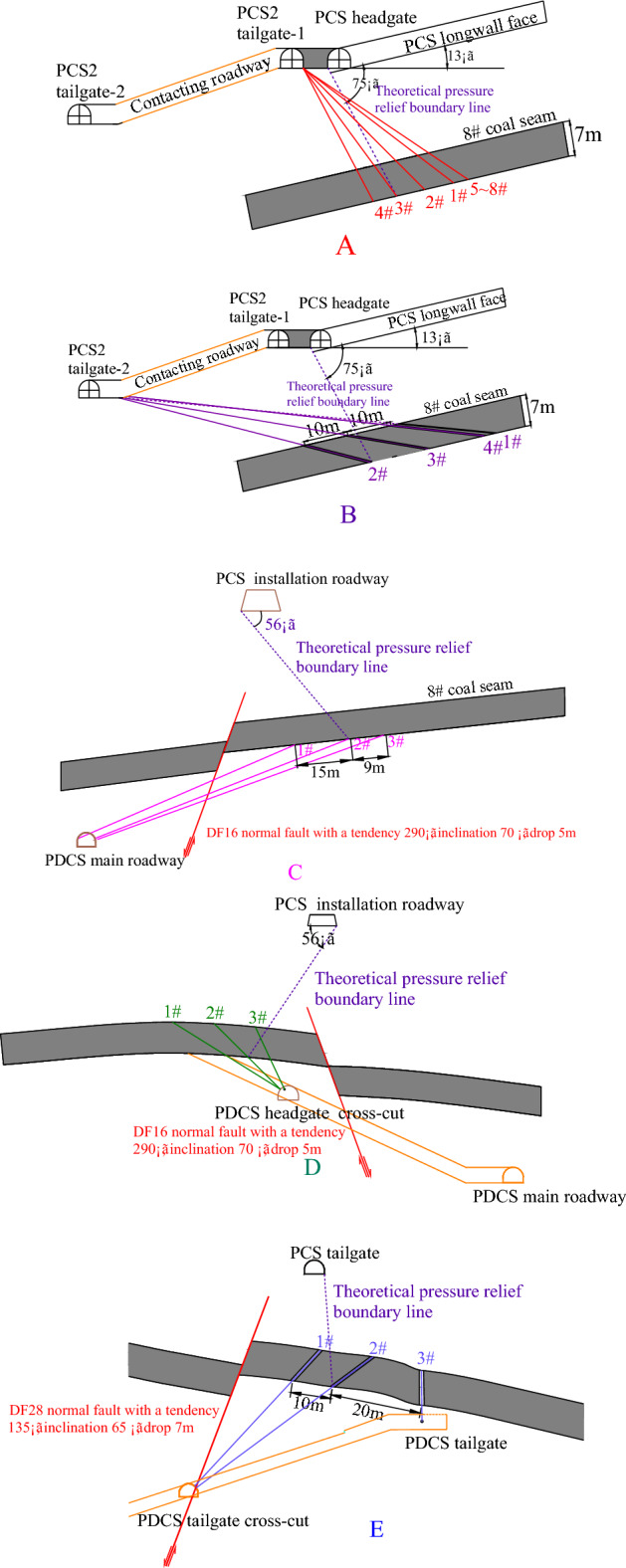
Design of the test drilling holes.

The dip angle of coal seams is 10–20°. According to the *provisions for the prevention of coal and gas outburst*^34^, the theoretical inclined and strike pressure-relief angle of the upper PCS mining is 75° and 56°, respectively. Therefore, locations of the test drill holes for gas pressure, content, and permeability coefficient should be around the theoretical pressure relief boundary (TPRBL), as shown in Fig. 5. Five groups (A, B, C, D, E) of test drills and their locations were theoretically designed and are shown in Figs. 5 and 6, respectively.

#### Test holes at the PCS2 tailgate-1 (Group A)

Holes were drilled into the PDCS longwall face in the tailgate-1 of the PCS2 longwall face before the PCS longwall face retreated. The PDCS longwall face will be mined after the PCS longwall face. The gas pressure of the PDCS longwall face was measured as the PCS longwall face advances. There were four test points along the strike and inclined of the PDCS longwall face, respectively, as seen in Fig. 6a. By testing the residual gas pressure in the 1^#^, 5^#^, 6^#^, 7^#^, and 8^#^ holes, it is possible to examine the pressure-relief effectiveness of the 8^#^ coal seam before and after mining of the PCS longwall face. However, because of the stress concentration of the surrounding rock during the PCS longwall face mining, the tailgate-1 of the PCS2 longwall face could not be excavated in time; therefore, the test holes in the tailgate-1 of the PCS2 longwall face were failed, so measurements are not available in these holes.

#### Test holes at the PCS2 tailgate-2 (Group B)

Due to the ventilation requirements, the tailgate-2 was constructed on the PCS2 longwall face, as shown in Fig. 5. There are four test holes in the tailgate-2 to observe the change in gas pressure and permeability coefficient in the PDCS longwall face during the PCS longwall retreating. The layout of the drilled holes is shown in Fig. 6b. As can be seen in Figs. 5 and 6b, the 2^#^ drill hole is located outside the pressure-relief scope at a distance of 12 m from the TPRBL; the 3^#^ drill hole is near the TPRBL at a distance of 2 m to the boundary; the 4^#^ drilling hole is located within the TPRBL at a distance of 8 m to the boundary.

#### Test holes at the PDCS main roadway (Group C)

After finishing the PCS longwall face, three test holes were drilled in the main roadway in the PDCS mining area. As seen in Fig. 5, the 3^#^ drilling hole was located within 10 m of the TPRBL, the 2^#^ drilling hole was located within 1 m of the TPRBL, and the 1^#^ drilling hole was located 14 m outside the TPRBL. Figure 6c shows the sectional view of the drilling holes. Because of the faults in this area, coal samples were not obtained during the construction of the 3^#^ drilling hole. The other two drilling holes were constructed according to the design angle. The measured gas content of the 1# and 2# holes is 8.71 and 5.39 m^3^/t, respectively. The gas content of the 2^#^ drilling holes decreased by 62.96%. However, there is limited pressure-relief in the 1# holes according to the measured gas content. The gas pressure measured results in Table 3 also shows only a 17.83% decreased.

**Table 3:** Field measurements of the gas pressure and gas content after protective coal seam mining.

Group	Drilling holes	Investigation site	Gas pressure (MPa)	Gas content (m^3^/t)	Permeability coefficient (m^2^/(MPa^2^ d))	Decrease in gas pressure (%)
B	2^#^	PCS2 tailgate-2	0.76	8.73^a^	0.008	17.64
	3^#^		0.50	6.88^a^	0.061	35.09
	4^#^		0.22	4.29^a^	0.792	59.53
C	1^#^	PDCS main roadway	0.75	8.71	0.008	17.83
	2^#^		0.33	5.39	0.164	49.15
D	1^#^	PDCS headgate cross-cut	0.18	3.94	0.760	62.83
	2^#^		0.30	5.85	0.148	44.81
	3^#^		0.52	6.72	0.011	36.60
	4^#^		0.14c	3.41	Failed	67.83
	5^#^		0.31c	5.28	Failed	50.19
	6^#^		0.49c	6.84	Failed	35.47
E	1^#^	PDCS tailgate cross-cut	0.69	8.59	0.008	21.79
	2^#^		0.35	5.23	0.178	46.89
	3^#^		0.27	4.69	0.898	54.81

^a^Using Eq. (9) to calculate the gas content or gas pressure.

#### Test holes at the PDCS headgate cross-cut (Group D)

**Figure 7:**
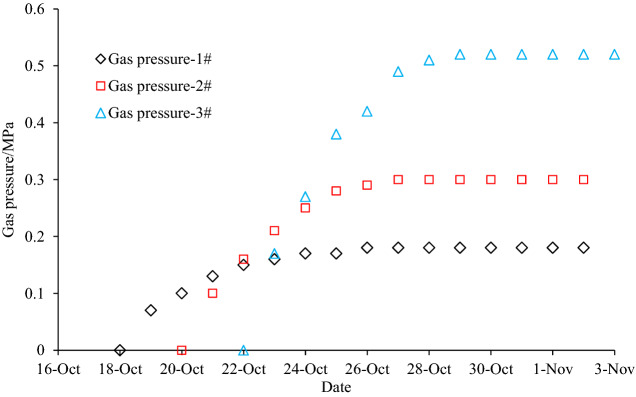
Gas pressure after protective coal seam mining.

Six test holes were designed at the headgate cross-cuts to measure the residual gas pressure in the PDCS and inspect the strike and inclined pressure-relief angles of the PCS longwall face. In Fig. 5, the 1#–3# drilling holes were used to test the strike pressure-relief angle and Fig. 6d shows the profile of the arrangement. The 1# hole was located within 10 m to the TPRBL; the 2# drilling hole was located on the TPRBL; and the 3# drilling hole was located 10 m outside the TPRBL. The 4#–6# holes were used to determine the inclined pressure-relief angle. The 4# hole was located within 10 m to the TPRBL; the 5# hole was located on the TPRBL; and the 6# drilling hole was located 10 m outside the TPRBL. As the 4#–6# holes were close to the PCS headgate, fractures around the holes developed. After the holes were sealed, a leakage occurred in the roadway; thus, the gas pressure and permeability coefficient measurements were not available. Therefore, only the gas pressure measurements in the 1#–3# drilling holes were implemented. The coal sample collection was executed in the 1#–6# holes, and the samples were used to test the coal seam gas content. The corresponding results are shown in Table 3. The gas pressure of the 1#–3# drilling holes is shown in Fig. 7. It clearly shows the gas pressure gradually increases but is still lower than the original gas pressure in the coal seams, indicating that there is also a certain pressure-relief beyond the TPRBL. Moreover, the further inside the TPRBL, the higher the pressure-relief.

#### Test holes at the PDCS tailgate cross-cut (Group E)

Three test holes were arranged in the PDCS tailgate cross-cut. The section and planar graphs of the arrangement are shown in Fig. 5. The 1# hole was located 10 m beyond the TPRBL; the 2# hole was located on the TPRBL; and the 3# hole was located within 20 m of the TPRBL. The gas content and permeability coefficient are listed in Table 3. The gas content from the 2# and 3# holes decreases to 61.09% and 54.78% of their original values, respectively.

## Field test results

### Gas pressure evolutions

**Figure 8:**
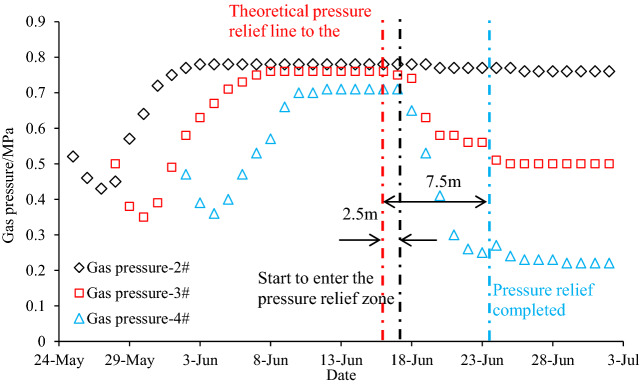
Gas pressure during protective coal seam mining.

If the monitoring area is located in the pressure relief zone, the decrease of coal seam stress leads to the increase of permeability, which further leads to the release of coal seam gas, and the gas pressure gradually decreases. Figure 8 shows the development of the gas pressure at each test hole of group B with the advancement of the PCS longwall face. The gas pressure gradually decreases when the PCS longwall face passes the monitoring point. The gas pressure at the 4# hole within the pressure-relief range has the greatest reduction; the gas pressure at the 3# hole located on the pressure-relief line also drops, indicating that the PDCS located around the 3# monitoring point also experienced pressure-relief but with a lower reducing rate of 34.2%. The result also demonstrates that the inclined pressure-relief scope is larger than that of the theoretical one, but the 3# hole only underwent partial pressure-relief comparing with the 4# monitoring hole. The gas pressure at the 2# monitoring point is unchanged, indicating that the pressure-relief effect is limited. Gas pressure measurement in the 1# hole failed, so the data is not available.

The gas pressure evolution showing in Fig. 8 indicates the strike pressure relief angle is smaller than the theoretical value. The gas pressure decreased rapidly 2.5 m behind the theoretical pressure relief line, and then remained constant until 7.5 m behind the theoretical pressure relief line. Therefore, it concludes the measured pressure relief boundary should be 2.5 m smaller than the theoretical one, and the corresponding strike pressure relief angle is 52.2°. Most of the pressure-relief gas will flow into the PCS longwall face through the floor fractures, which affects the safety of PCS mining^11^. Therefore, gas drainage using floor drillings is recommended to extract the pressure-relief gas during the PCS longwall face retreating. It is helpful to reduce the gas pressure in the pressure relief range, as well as to expand the pressure relief range in the PDCS.

### Permeability coefficient evolutions

**Figure 9:**
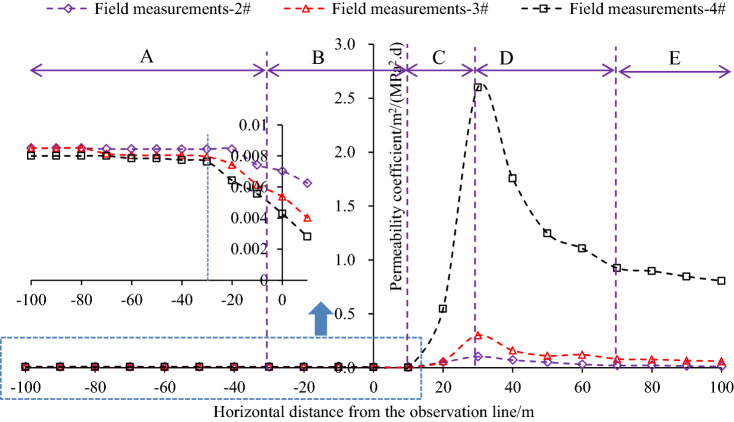
Permeability coefficient evolution during protective coal seam mining. A is In-situ stress zone, B is abutment stress influence zone, C is significant pressure-relief zone, D is gradually compacted zone, and E is compacted stable zone. In the figure, negative and positive of the x-axis mean the monitoring holes are located in front of and behind the longwall face, respectively.

**Figure 10:**
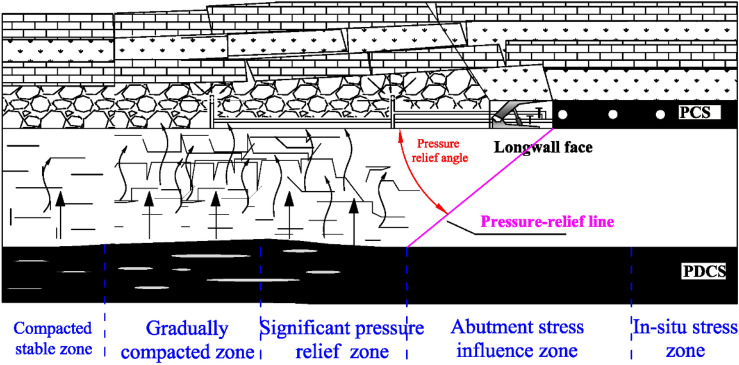
Schematic diagram of different pressure relief zones in PDCS during PCS mining.

Permeability coefficient is monitored in the 2#, 3#, and 4# holes in group B after gas pressure measurements. The monitored results during the PCS retreating are shown in Fig. 9. The mining of the PCS causes a significant increase in the permeability of the PDCS. The permeability evolution in each monitoring holes can be divided into four stages: slowly decreasing, sharply increasing, gradually decreasing, and basically stable. Taking the monitoring data of 4# borehole as an example, the permeability of the PDCS remains if the monitoring location is beyond the effect of the PCS longwall mining, i.e., more than 30 m away from the longwall face. The permeability slowly decreases when the boreholes in the range of − 30 m and 10 m in relation to the longwall face. It then increases sharply with a distance of 10 m to 30 m behind the longwall face. After that, the permeability dramatically reduces until the monitoring holes are approximately 70 m behind the longwall face. Finally, the permeability gradually stabilizes. The permeability evolution attribute to the stress change of the PDCS during the PCS mining, as shown in Fig. 10. The PDCS can be divided into five zones during mining of the PCS: the in-situ stress zone, the abutment stress influence zone, the pressure-relief zone, the gradually compacted zone, and the compacted stable zone. In the in-situ stress zone, the mining of the PCS does not affect the PDCS, and the permeability unchanged. In the abutment stress influence zone, the stress increase causes the permeability to decrease gradually. While in the pressure relief zone, a large number of expansion fractures are created, significantly increasing the permeability. In the gradually compacted zone, the permeability gradually decreases as the goaf compacts. When the goaf is compacted, the permeability of the PDCS remains constant during the monitoring period.

In Fig. 9, it can be seen that the permeability has relatively large enhancements in the pressure relief zone (~ 20 m in length), the gradually compacted zone (~ 40 m in length) and compacted stable zone. The maximum permeability coefficient is 2.58 m^2^/(MPa^2^ d), which is about 322 times the original permeability (0.008 m^2^ /(MPa^2^ d)); the minimum permeability coefficient in the compacted stable zone during the monitoring period is 0.81 m^2^/(MPa^2^ d), which is about 101 times the original permeability. It shows that the permeability coefficient still stays at a high level even if the stress recoveries to the in-situ state^28,35^. Therefore, it is the best choice for gas extraction within this area.

**Figure 11:**
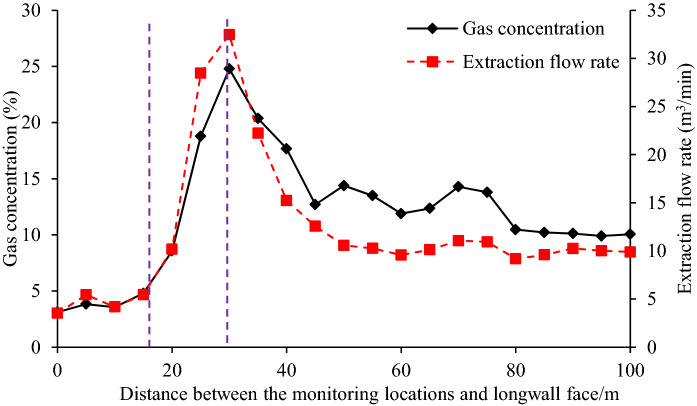
Gas extraction flow rate and gas concentration in the high roof roadway drainage in PDCS.

The evolution of gas extraction rate and concentration in the PDCS during PCS advancing are shown in Fig. 11. When the monitoring holes were 15 m behind the PCS longwall face, the gas extraction rate and concentration begin to increase significantly. The two arguments maximize at the location about 30 m behind the longwall faces, and then gradually declines to constant values. The evolution of two arguments further verifies the permeability measurements, showing the zonal characteristics and pressure relief dependency. It should be noted that the decrease in gas extraction rate is not only related to the permeability decrease but also resulted from the reduction in gas pressure^2,26^.

To study the pressure relief scope, the evolution of permeability coefficient in 3# and 2# boreholes located outside of 4# borehole is also given in Fig. 9. The permeability coefficient at the 3# monitoring point (located on the pressure-relief line) also increases, indicating the PDCS also has a pressure-relief effect near the pressure-relief boundaries with the maximum permeability coefficient of 0.3 m^2^/(MPa^2^ d). While outside of the pressure-relief range, the pressure-relief effect is limited. For example, the maximum permeability coefficient of 2# borehole reached 0.102 m^2^/(MPa^2^ d) (12.75 times the original value); however, the permeability coefficient quickly decreases to a low level due to the short time of the pressure relief effect.

## Determination of the pressure relief angles and gas outburst control area

Because the monitoring boreholes in groups C, D, and E are close to the installation roadway, it is difficult to monitor the evolution of gas pressure and permeability during the longwall face retreating. Moreover, continuous monitoring is relatively complicated and high cost. Therefore, continuous monitoring was only carried out in group B boreholes. The residual gas content, pressure, and permeability coefficient of the PDCS were measured after the PCS longwall longwall face finished.

In the process of drilling, some boreholes only took out the coal samples, but the gas pressure was not measured. Besides, since the coal sample needs to be taken out during the gas content measurement, the same borehole can only be measured once. For continuous monitoring, the borehole in group B has been drilled before PCS mining; thus, the measurement of residual gas content cannot be implemented. According to the results of the gas pressure or gas content test, and considering the adsorption constant of 8# coal seam (see Table 1), the gas content or gas pressure in each hole can be calculated by Eq. (9) ^36^.

9 $$ W_{CY} = \frac{{ab\left( {P_{CY} + 0.1} \right)}}{{1 + b(P_{CY} + 0.1)}} \times \frac{{100 - A_{ad} - M_{ad} }}{100} \times \frac{1}{{1 + 0.31M_{ad} }} + \frac{{\pi \left( {P_{CY} + 0.1} \right)}}{{\gamma P_{a} }} $$

where *W*_CY_ is the residual gas content (m^3^/t); *P*_CY_ is the residual gas pressure (MPa); *a* and *b* are adsorption constants (mL/g and MPa^−1^, respectively); *π* is the coal porosity. The calculated gas content in group E is 8.27, 5.60, and 4.82 m^3^/t, respectively. The errors compared with the measured data were 3.7%, 7.1%, and 2.8%, respectively, indicating that Eq. (9) could provide convincing calculations. The calculated gas pressure and gas content are shown in Table 3.

### Determination of pressure relief angle

#### Strike pressure-relief angle

**Figure 12:**
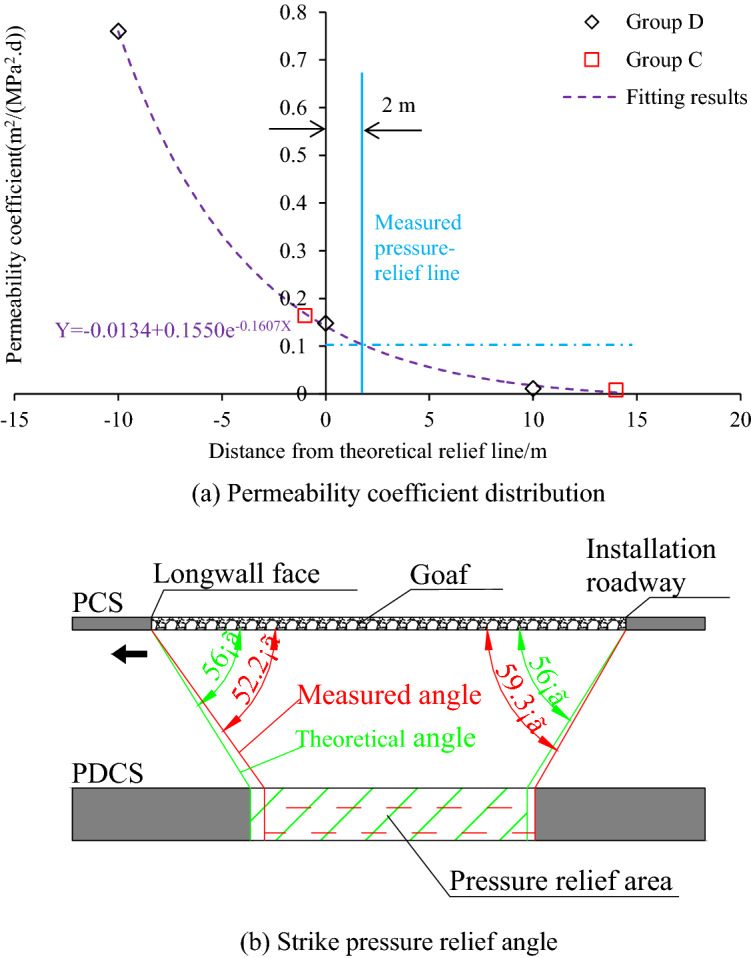
Strike pressure relief angle and its calculation.

The strike pressure-relief angle is generally divided into two sides, as shown in Fig. 12b. One side is at the active longwall face, and the other is at the installation roadway. Since the longwall face is advancing along the strike (i.e., no inclination angle), the two angles on both sides are the same. According to the evolution of gas pressure in group B boreholes, the measured strike mining pressure-relief angle during the longwall face advancing is 52.2°, which is smaller than the theoretical one (56°). Gas extraction is carried out on the PDCS, which can accelerate the reduction of gas pressure and content, and expand the pressure-relief scope near the installation roadway. Therefore, the permeability coefficient is used to determine the pressure-relief angle. According to the changes in the permeability coefficient monitored by group C and group D, the permeability coefficient decreases gradually from inside to outside with a negative index. An extractable critical permeability coefficient of 0.1 m^2^/(MPa^2^ d) was used as a judgment index of effective pressure relief range according to *Coal Mine Safety Regulations*. The measured effective pressure relief range extends 2 m from the theoretical line (Fig. 12a), and the corresponding pressure-relief angle is 59.3°, a little larger than the theoretical one (56°), as shown in Fig. 12b. The pressure-relief angle at the installation roadway is higher than that at the active longwall face side, which may result from the following two reasons: (1) the pressure-relief angle at the longwall face is monitored in real-time by borehole group B, which is a dynamic parameter, and lag behind the stress transfer process. However, at the installation roadway side, the angle was measured after sufficient pressure relief occurred. (2) the decrease of gas pressure also increases permeability^26^, which caused further expansion of the pressure relief range. Therefore, the strike pressure relief angle at the installation roadway is larger than the dynamic one at the longwall face side.

#### Inclined pressure-relief angle

**Figure 13:**
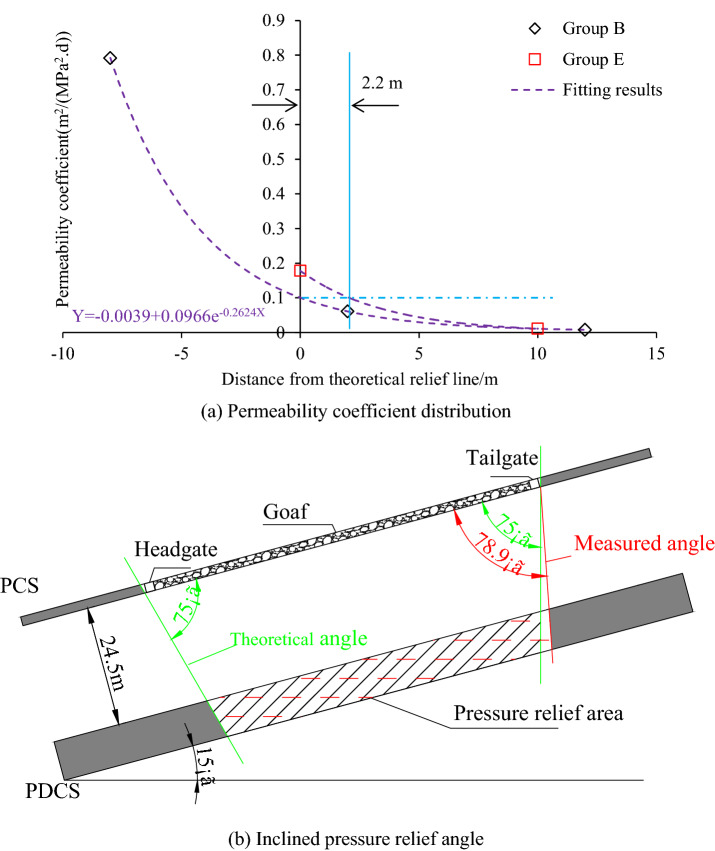
Inclined pressure relief angle and its calculation.

The inclined pressure-relief angles at each side of the longwall face may differ due to the inclined angle (15°) of the coal seam. The boreholes in group B and group E are used to determine the inclined pressure-relief angles on each side of the longwall face, as shown in Fig. 13. 1# and 2# boreholes in group E are used to calculate the pressure-relief angle at the tailgate side. As shown in Fig. 13a, the measured pressure relief line on the headgate side coincides with the theoretical relief line, and the corresponding pressure-relief angle is 75°. In contrast, the pressure relief line on the tailgate side expands outward by 2.2 m, and the pressure relief angle is 78.9°, as shown in Fig. 13b. Therefore, the upper side (tailgate side) witnesses a better pressure relief effect, which results from the higher recovery goaf stress at the bottom side (headgate side) of the longwall face^37^.

### Determination of gas outburst control area

**Figure 14:**
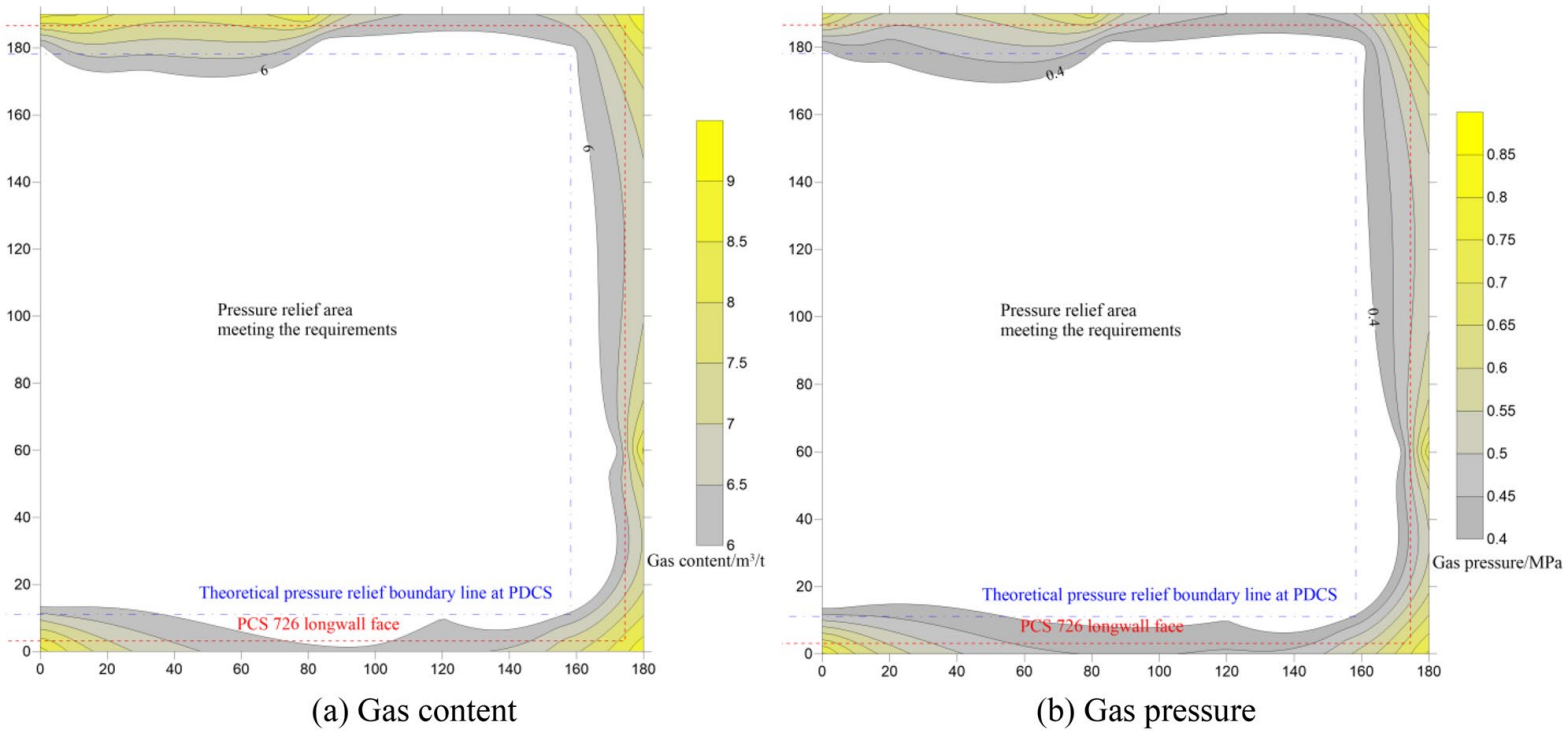
Distribution of the gas content (**a**) and residual gas pressure (**b**).

The above analysis determines the pressure-relief scope in the PDCS, but the coal seam in the pressure-relief zone does not mean removing the outburst danger. According to the requirements of *Coal Mine Safety Regulations*^9^, the gas content in the coal seam shall not be more than 6 m^3^/t if the caving mining method is employed. The gas pressure is estimated to be 0.40 MPa, according to Eq. (9). The distribution of the measured residual gas content and residual gas pressure in the PDCSis shown in Fig. 14 (also see Table 3).

The gas content of the PDCS in the pressure-relief area is reduced to less than 6 m^3^/t (minima of 4 m^3^/t). The corresponding gas pressure is usually less than 0.4 MPa (minima of 0.18 MPa) in the pressure-relief area . However, outside the pressure-relief area, both of the two parameters also decrease mainly due to the gas extraction during the PCS mining. With away from the PCS pressure-relief zone, the two arguments gradually increase to their original values. Therefore, field measurements show that pressure-relief mining can remove the risk of coal and gas outbursts in the PDCS. There was no gas overrun and outburst accident during the mining process of the PDCS, which further confirmed the effect of pressure relief mining.

## Discussions

**Figure 15:**
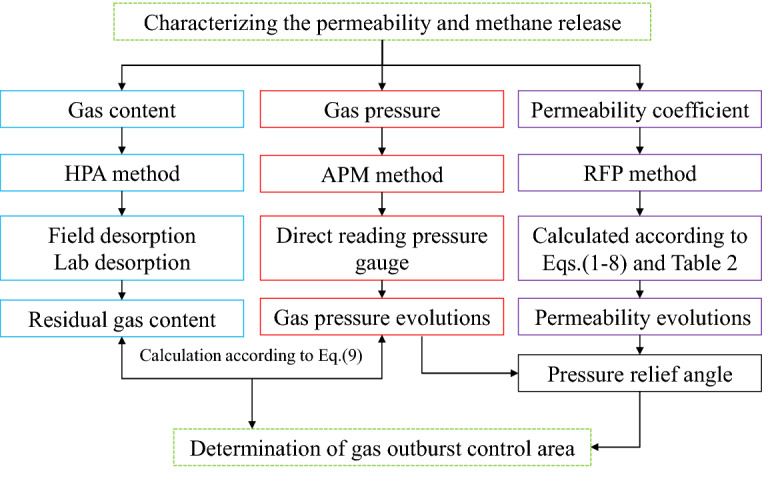
Field measurement flow chart for Characterizing the permeability and methane release.

Pressure-relief mining is the fundamental method to remove coal and gas outbursts in deep, high-gas pressure, and low-permeability coal seams. For the safe and efficient extraction of the subsequent PDCS, the effect of pressure-relief mining and the pressure-relief area in the PDCS were evaluated by field measurements in this paper. Because of the high cost, delicate operation, and danger of field measurements, it is usually challenging to monitor and evaluate the effectiveness of the pressure-relief of PCS mining. Besides, field measurements are greatly affected by mining activities and always have a low success rate. Therefore, most field measurements focused on monitoring the residual gas content or gas pressure of the PDCS after the PCS mining, which cannot fully characterize the evolution of these parameters. In this paper, by using intensive boreholes, detailed and whole mining process field measurement is carried out. For the first time, the evolution law of permeability and gas pressure in the mining process is obtained. The corresponding field measurement and calculation methods of gas pressure, gas content, and permeability coefficient are proposed and shown in Fig. 15. Considering the numerical results are poorly verified by field applications, doubting the reliability of the simulations. The field observations in this paper can serve as benchmark evidence for theoretical analysis and numerical simulations.

## Conclusions

1. The measured results show that the gas pressure of the PDCS decreases after the PCS longwall face advances a certain distance from the monitoring point due to the existence of a pressure relief angle. The gas pressure in the PDCS outside the pressure-relief range is unchanged. The inclined pressure-relief angles of the PCS mining at the active longwall face side and installation roadway side are calculated to be 52.2° and 59.3°, respectively.
2. The permeability coefficient evolution in the pressure-relief range of the PDCS can be divided into four stages: slowly decreasing, sharply increasing, gradually reducing, and basically stable. The corresponding four stress zones in PDCS are the abutment stress influence zone, the pressure-relief zone, the gradually compacted zone, and the compacted stable zone, respectively.
3. The PDCS has the best permeability enhancement in the pressure-relief zone and the gradual compaction zone, with the length of 20 m and 40 m, respectively, and the maximum permeability is 322 times of the initial permeability. The final permeability coefficient in the compacted stable zone during the monitoring period can reach 100 times of the initial permeability. The permeability in the regions located outside the pressure -relief boundary, can also be improved to a certain extent when entering the pressure-relief zone but returns to the initial value after it compacted.
4. The inclined pressure relief angle at the lower end of the inclined longwall face is 75° consistent with the theoretical pressure relief angle; the measured pressure-relief angle at the upper end of the air roadway is extended to 78.9°.
5. The residual gas content and residual gas pressure of the PDCS in the pressure-relief area are reduced to less than 6 m^3^/t and within 0.4 MPa, respectively. These two parameters also decreased outside the pressure-relief zone, albeit not significantly. The field measurement results in this paper further prove that pressure-relief mining can prevent coal and gas outbursts in PDCS.

## Data Availability

Some or all data, models, or codes that support the findings of this study are available from the corresponding author upon reasonable request.
